# Synopsis of the Proceedings of the Macon Medical Association, Augt. and Sept.

**Published:** 1871-11

**Authors:** 

**Affiliations:** Macon, Ga.


### SYNOPSIS OF THE PROCEEDINGS OF THE MACON MEDICAL ASSOCIATION, AUGT. AND SEPT.

MACON MEDICAL ASSOCIATION,
MACON, GA., AUGUST 1871.

The Association met and proceeded to business, with its President, Dr. C. B. Nottingham, in the Chair.

Written communications being called for, in order, Dr. Wm. R. Burgess, the Essayist for the evening, presented and read a paper on quinine, considered only under its repeated emmenagogue properties. He seeing the subject was being agitated throughout the country, and the professional mind being somewhat divided in regard thereto, a medical friend had, some twelve months ago, suggested to him the importance of noting, with care, the result of administering quinine in periodical doses to such pregnant females as might fall under his treatment. In obedience therefore, to this suggestion, he reported several cases of malarial fever in pregnant women, when the remedy was freely and fully used without any deliterious after-consequences, but *per contra*, when uterine contractions were giving serious trouble, threatening even the expulsion of its contents, so soon as the system could be impressed and the fever arrested, these symptoms were invariably set aside. Closing his report of cases, he said: "I might mention other cases where I gave quinine freely, to patients during the time of their illness, but as these are all in which a special note was kept, I will allude to no others. Suffice it to say, that I have never witnessed a case where I believed quinine induced premature expulsion of the uterine contents. Yet it will be borne in mind that in none of the cases reported, was more than 20 grains given in twenty-four hours, and this quantity were given at a single dose. Ten grains being the largest amount given at any one time. Still my patients were frequently impressed to the extent of annoying deafness and tingling of the ears. The effect of 20 or 30 grains, administered at a single dose, might be different; but as I rarely recognise the necessity for such "heroic" dosings without further cause for changing my opinion, I must look upon quinine as an invaluable agent in the management of the class of troubles now under consideration."

Further he says, "I am neither ignorant nor unmindful of the fact, that physicians of our own town, of large ability and experience, entertain an opposite opinion; and with this fact in view, my mind has been the more closely drawn to the observance of the cases reported above. Little however, is ever attained by unverified opinion, and while I entertain the highest possible regard, both personal and professional, for my co-laborers, whose opinions stand opposed to my own, I know of no better plan to arrive at facts, (which are emphatically what we want,) than to array the tesmony on both sides, and draw conclusions." In closing his paper, for the purpose of arriving at the voice of the profession, he invited free criticism, when the following discussion, substantially, occurred:

Dr. Geo. N. Holmes being called on, said: "Mr. President, I endorse fully the remarks of Dr. Burgess. It has been my practice, all my life, to administer quinine to pregnant women, when called to treat them for such diseases as indicate the use of antiperiodic medicines, and I have never witnessed a single case where abortion or miscarriage resulted from its use."

Dr. Chas. H. Hall remarked: "I fully agree with Dr. Burgess. Sixteen years of practice has not given me one instance of injury to the pregnant woman. As early as 1856, I had recorded cases of pregnancy in which the drug was given with special reference to satisfying my mind on this question. That record agrees with

Dr. B's."

Dr. W. F. Holt, said: "Mr. President, I am constrained to take grounds opposed to those who have preceded me in this discussion. I feel sure that I have witnessed one or more cases of premature parturition conclusively referable to the administration of quinine. So fully impressed have I been with this conviction, that it is my invariable rule, when deeming it necessary to resort to the use of the medicine in the treatment of pregnant women, to combine with it some form of opiate."

Remarks of Dr. P. H. Wright: "The question before us to-night for discussion, 'will quinine, when combined with opium, produce abortion in the pregnant female?' being one of the greatest importance, both to the pregnant woman and the physician, I deem it very essential that there should be a *free* and *full* expression of opinion by all present, and for one, I will be glad to hear the honest opinion of every member of this Association fully expressed to-night. I will then, in the beginning, state most positively, that I am decidedly of opinion that quinine, even uncombined with an anodyne, possesses no tendency, whatever, to produce abortion.

In the beginning of my professional life, in Louisianna, on the banks of the Mississippi River, where quinine is the drug—*par excellence*—and is given almost ad libitum, both by the profession and the people, I was in blissful ignorance of even a suspicion, that it had any such powers, and therefore, gave it freely to pregnant women, and I cannot now recall to mind a single instance where it ever produced abortion in my own practice, nor do I think it did so in the hands of the overseers on those large plantations, who gave it daily, and indiscriminately, to *all*. Abortions were seldom ever among those women who were worked hard on the plantations. Since then. and during my residence in this city, I have had frequent occasions to give quinine to pregnant women, and cannot now recollect, a single instance in which it seemed to exercise such a tendency. I have, within the past month, had occasion to administer the drug alone, in from 12 to 15 grain-doses, daily to a delicate lady, four months advanced in pregnancy, who was suffering from an attack of intermittent fever, and in this case there was not the slightest evidence that the uterus was even apprised of the presence of quinine in the circulation. The lady is now well, and carrying the fœtus comfortably. We all know, and I belive it is universally considered, that frequent attacks of chills and fever, or billious remittent fever, do tend to produce premature labor, and with all due difference to the opinions of those gentlemen who differ from me on this all-important question, I believe that to withhold quinine under such circumstances, is to subject the patient to the most serious danger of miscarriage. In my opinion then, we should direct our treatment to subdue the disease, and thereby prevent abortion. Again, I have very serious doubts whether quinine produces any emmenagogue properties primarily, and if it does secondarily, it is due solely to its tonic prophylactic properties, and this may be said of any other drug which tend to build up the enfeebled system.

In conclusiou then, I would say that I do not hesitate to administer quinine to any pregnant female, in any stage of pregnancy, whenever I believe that it is required. Lastly, I don't believe that quinine will produce abortion. I believe its tendency is rather to prevent it, by arresting the exacerbations of chills and fever. I do not believe it is emmenagogue, except it may be secondarily through its tonic and prophylactic properties."

Remarks of Dr. A. L. C. Magruder: "I have practiced medicine for thirty years in malarial districts, where the use of quinine was constantly indicated in the treatment of disease. During that period I have prescribed it frequently for pregnant females, and in no case, to the injury of the patient, when abortion or premature labor was threatened from fever, the symptoms of labor have been immediately arrested by the cure of the fever. In such cases however, owing to a prevailing opinion of the emmenagogue properties of quinine, I have almost always guarded the use of quinine with some opiate."

Remarks of Dr. C. B. Nottingham: "I have felt deeply interested, gentlemen, in the discussion of the evening, believing as I do, the subject to be greater, of more grave, importance than any other question of therapeuctics that has ever engaged the attention of this body in the free enterchange of views, upon which there has been developed a radical difference of opinion. The propriety of administering quinine to the pregnant woman when viewed in its relations to the comfort and safety of the mother, the life of the child, the happiness of families and the interest of State, is of profound interest, and I am glad to say that my observation and experience has been entirly in consonance with the views and general tenor of the very interesting paper read by the Essayist of the occasion, Dr. Burgess.

Accustomed, all my professional life, to administer quinine as freely to pregnant females as to any other class of persons requiring its antiperiodic, antidotal or tonic influence, I do not recollect a solitary instance in which I have had the least reason to suspect it of having been disastrous in the production of abortion or miscarriage. It is true, these untoward events have sometimes occurred with patients whilst taking it, but I have uniformly attributed them to the shock of chills, the disturbing influence of fever, the broken and anæmic condition of the system, or to some circumstance antidating the illness, and not in any case to the quinine. Neither has it been my habit to combine opium or any of its preparations with the quinine in this class of patients. If the state of the stomach, the condition of the bowels, or the existence of nervous phenomena call for opium, I give it, but not otherwise, as I do not recognise any parturient property in quinine that reqiures opium or other annodyne to hold it in check.

As a direct emmenagogue, I neither attach value to or entertain fears of quinine. If possessed of such power it has entirely escaped my observation, and I have been in the habit of constantly giving it alike to the victims of ammenorrhœa and to menstruous females when laboring under malarial fever, periodic neuralgia and various other maladies, calling for its use.."

TUESDAY EVENING, SEPT. 5TH, 1871.

The Association met, etc. The discussion on quinine continued from last meeting. Dr. D. W. Hammond, the Essayist of the evening read a paper on the subject, of which the following is a synopsis: "The question for discussion this evening, is a very important one. Does quinine predispose to abortion, in antiperiodic doses, in remittent fever? Does it possess emmenagogue properties? I generally prescribe it for pregnant women in intermittents, and abortion has frequently taken place, whether this was the result of the toxic effect of the quinine, or malarial poison, I am not fully satisfied in my mind. I am of the opinion that the violent fever following malarial intoxication may cause miscarriage, independent of the quinine. When a pregnant woman has had chills and fever for a considerable time, the result is great prostration and anasarca. In such cases abortions often follow, but in these cases quinine has been liberally administered. Again, many pregnant females endure chills for months from a fear of quinine, from which the red corpuscles of the blood are broken down and impoverished from malarial poison, and have gone to the full term. Then to say that quinine produces arbortion, or that malarial poison is the cause, is to guess at it. Who can say positively which caused the accident? I am of the opinion that quinine is a partus accelerator, and that it possesses emmenagogue properties, if not directly, certainly indirectly.

I acknowledge the weight of authority is against me on this subject. Here the Dr. read several interesting communications from distinguished members of the profession, in regard to the action of quinine upon pregnant women: Dr. Arnold, of Savannah, Dr. Eve, of Augusta, Dr. Bevins, of New Orleans, Dr. Wallace, of Jeff. Med. College, of Philadelphia, and Dr. Byford, of Chicago.

All of whom, with singular unanimity, testify that, according to their experience and judgment, quinine is totally devoid of parturient powers, and if it possesses emmenagogue properties they are secondary through its tonic influence. It would be interesting to give the entire letters of these eminent gentlemen, but space will not allow it. Per-contra, the Doctor quoted from Billingslea and Wilson, of Alabama, Drs. Phares and Carruth, of Mississippi, Dr. Yandell, of Kentucky, and Dr. Mon Cuerde, of England, as entertaining opposite views.

Dr. J. Emmett Blackshear stated, that he had never hesitated to prescribe quinine for the pregnant woman, when its use was indicated; that he had never known abortion to occur during its administration, that could be attributed to the medicine; and that he considered it neither a parturifacient nor an emmenagogue.

The discussion was extended during the evening, by gentlemen whose remarks, as made at a previous meeting, have already been given.

